# Positional relationship between ball and fingers for accurate baseball pitching

**DOI:** 10.1371/journal.pone.0290042

**Published:** 2023-12-19

**Authors:** Ayane Kusafuka, Kohei Nishikawa, Naoki Tsukamoto, Kazutoshi Kudo

**Affiliations:** 1 Department of Intermedia Art and Science, Faculty of Science and Engineering, Waseda University, Tokyo, Japan; 2 Department of Life Science, Graduate School of Arts and Sciences, The University of Tokyo, Tokyo, Japan; Opole University of Technology: Politechnika Opolska, POLAND

## Abstract

Accurately throwing an object to a target position repeatedly is one of the specific human motor skills. The final arrival position of a thrown ball can be determined by its physical state at release. In baseball pitching, reducing the variability of the velocity angle of the ball at release (release angle) is important for reducing the variability of the pitch location. Although previous studies have suggested that hand and finger movements are important for accurate throwing, their relationship with the release angle has not yet been investigated in detail. This study focused on the positional relationship between the ball and fingers, which is considered to be closely related to ball movement as an action point of the force, and examined its relationship with the variability of release angle. To obtain accurate finger positions relative to the ball without impeding movement or sensation, an automatic image recognition technology based on deep learning was employed. This approach revealed a noteworthy correlation between the lower middle finger positions prior to acceleration peaks and the reduced variability in release angle, emphasizing the importance of consistent finger positioning during the pre-release phase. This finger positioning of the pitchers with low variability in the release angle is suggested to be robust against the spatial variability of ball movement.

## Introduction

Accurately controlling an object and repeatedly throwing it to a specific target position is a fundamental human motor skill. This skill is prominently demonstrated in a variety of sports, including baseball pitching, handball shooting, and dart throwing. The flight trajectory and arrival position of the ball can be determined by its physical state at release or impact when the environmental factors are constant. In baseball pitching, the aerodynamic interaction between the surface profile of the ball (e.g., seams) and the airflow around it (e.g., wind speed and atmospheric density), which cannot be controlled, acts on the ball [1, 2]. However, the nine mechanical parameters of the ball at release, referred to as the release parameters, are the main determinants of the pitch location [3, 4]. The release parameters include the position, velocity, and rotation of the ball, each of which has a different degree of influence on the variability of the pitch location [5, 6]. The reduction of the variability of the ball velocity angle at release, referred to as release angle, is reported to be a key factor in minimizing pitch location variability during fastball pitching [7]. The release angle is determined by the elevation and azimuth angles of the velocity in polar coordinates. Specifically, it represents the angle between the velocity vector in the sagittal plane and the sagittal horizontal axis, as well as the angle between the velocity vector in the horizontal plane and the sagittal horizontal axis. As their paper showed that the variability of the release angle directly influenced the variability of the pitch location, the mechanism involved in the reduction of the variability of release angle should be clarified for accurate pitching. However, the determination of the release angle, as well the body movements that should be performed to reduce the variability of the release angle are not clear.

Given that only the fingers come into contact with the ball just before release, it seems reasonable to assume that finger movements have a significant impact on all the release parameters including the release angle. It has been suggested that the timing of the finger joint extension is important for accurate pitch location when throwing from a sitting or static standing position [8, 9]. However, the finger movements during pitching have been treated as an integral part of the palm in most previous research [10–12]; a few recent studies have focused on the interphalangeal joint movements of the fingers and have finally clarified the kinematics (e.g., joint angles) and kinetics (e.g., joint torques) [13, 14]. Therefore, there is no clear knowledge of the influence of finger movement on the release parameters available in the previous literature.

Given the laws of physics, the release angle should depend on the force on the ball at release. This force is determined by three elements: magnitude, direction, and point of action. Mainstream pitching research has been able to back-calculate the magnitude and direction of the net force acting on the ball (the sum of the forces from the body) from the ball movements including the acceleration [13]. However, when studying ball control, the point of action should also be considered in terms of the possible elements that can be controlled by humans. Therefore, we focused on the action point, i.e., the positional relationship between the ball and fingers that touch the ball, rather than the interphalangeal joint movements, to reveal the factors that affect the release angle more directly. Moreover, due to the relatively low force exerted on the ball by the fingers during release, there is a delay before this force is translated into the velocity of the ball. Hence, our investigation focused not only the release moment of release but also the time series change of the positional relationship between the ball and fingers, aiming to elucidate its connection to the variability of the release angle. We hypothesized that the positional relationship between the ball and fingers before the moment of release influences the variability of the release angle.

## Materials and methods

### Experiment

Twenty skilled baseball pitchers (sex: male; age: 20.6 ± 1.9 years; height: 176.6 ± 4.8 cm; weight: 73.5 ± 8.6 kg; seventeen right-handed and three left-handed) without any current injury participated in the study. All the pitchers belonged to a top-level university baseball league in Japan. Their pitching styles included seventeen overhands and three underhands. All the experiments were performed in an outdoor field. The pitchers warmed up by performing light catch and pitching practice before the experiment. They pitched 30 four-seam fastballs to the mitt of a catcher (approximately 20cm in width and length), whose center was aligned 0.5 m behind the home plate (18.44m from the center of the pitcher’s plate) and 0.9 m above the ground. The pitchers were instructed to aim at the catcher’s mitt and throw as fast and accurately as possible.

**Fig 1:**
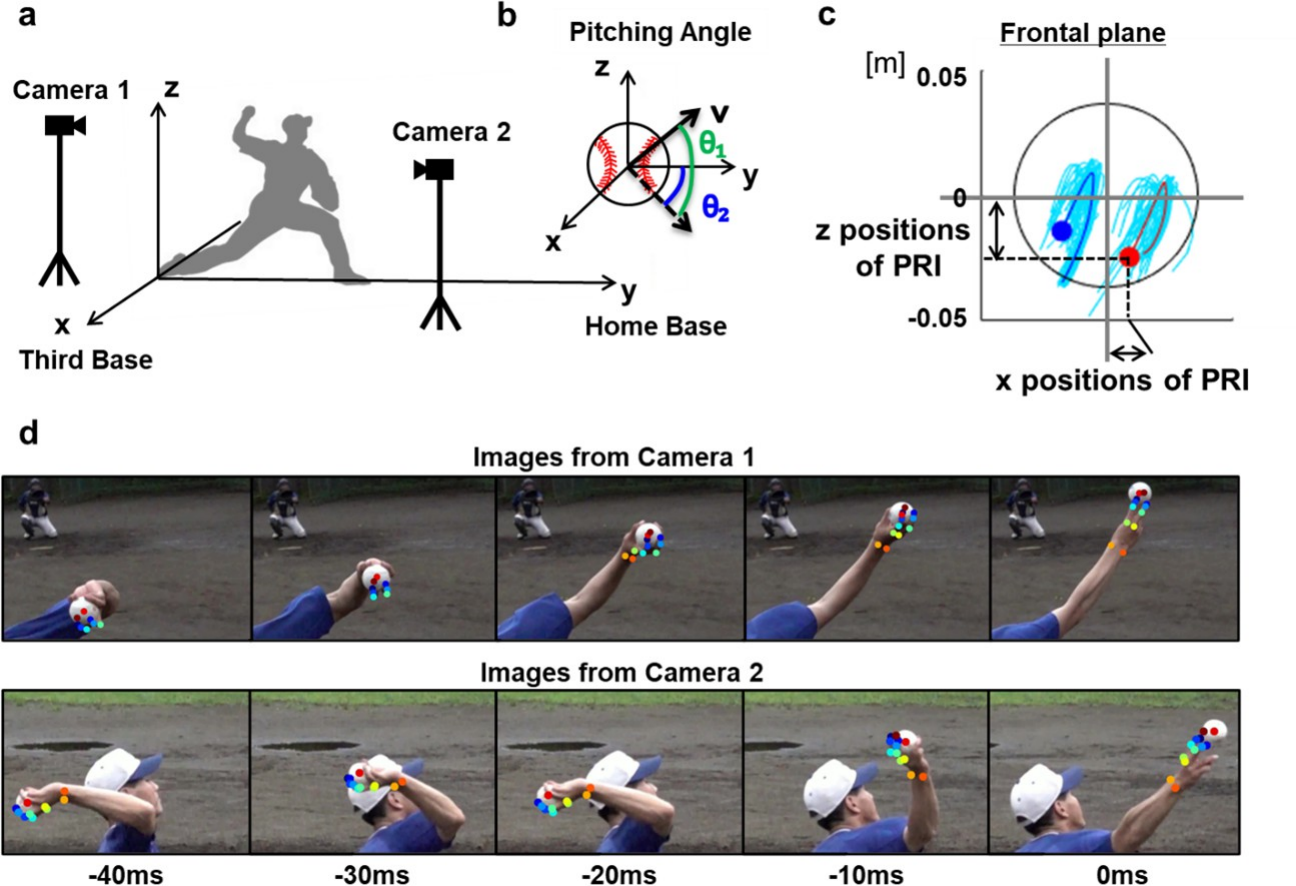
Three-dimensional orthogonal coordinate setup used in this study. (a) The x-axis extends from the first base to third base, the y-axis extends from the pitcher’s mound toward home base, and the z-axis runs vertically upward from the ground. The origin is the center of the plate on the mound. (b) The pitching angle is determined by the elevation angle θ_1_ (ranging from -90° to 90°) and azimuth angle θ_2_ (ranging from -90- 90°). (c) The image of PRI of middle finger. PRI was defined as the time series of the 3D positions of the nails on the index and middle fingers relative to the center of the ball in the global coordinate system. (d) The sequential images of the ball and fingers obtained by the two high-speed cameras.

The ball and finger movements were recorded using two high-speed cameras (DSC-RX10M4, SONY, Japan; 960 fps) placed on the back and side of the pitcher (Fig 1). The two cameras were synchronized using an optical light-emitting diode (LED) light and a commercial synchronization system (FA-WRC1M, SONY, Japan; FA-WRR1, SONY, Japan), which allowed the cameras to release the shutters simultaneously. The position coordinates were obtained from the centers of the metacarpophalangeal joint, proximal interphalangeal joint, distal interphalangeal joint, and nails of the index and middle fingers of the throwing hand, and from the center of the ball. No markers were placed on the body of the pitchers and centers of the ball. Consequently, the position coordinates of the fingers and the center of the ball were determined using the camera images. The movement phase for data acquisition was approximately 60 ms, from the maximum external rotation of the shoulder joint, to approximately 10 ms after ball release. The data for which the movement was out of the angle of view of the cameras because of the variability in movement between trials were excluded; hence, the data from three of the pitchers were not considered. Therefore, the following analyses were performed for seventeen pitchers. The study was approved by the ethics committee of the University of Tokyo and all the participants provided written informed consent.

### Data analysis

The digitized points on the ball and fingers in the high-speed camera images were obtained using an automatic image recognition technique based on deep learning, DeepLabCut [15]. Before training, the movies were trimmed to 60 frames (40 frames before ball release and 20 frames after). The ball release frames were chosen by qualitative observation of the camera images from the camera placed on the side of pitcher. The frames that indicated ball release were also ensured using the distance between the center of the nail of the middle finger and the ball calculated from the positional coordinate. We trained our models using labeled images from a trial of each pitcher. Twenty images from each trial were extracted using an automated feature in DeepLabCut which identified the optimized frames for manual labeling, yielding 340 manual labeled images for model training. After 35000 to 55000 repeated network trainings using these images, we used these models to track and digitize points on the images of all the remaining trials. A separate model was created for each camera. Trial data for which accurate points could not be obtained due to variability in weather-derived brightness between trials were excluded, and consequently, the data of three more pitchers were removed. Thus, the following analyses were performed on the data of fourteen pitchers. As the removed data included those of all the underarm pitchers, the fourteen pitchers (age: 19.8 ± 1.0 years; height: 174.8 ± 3.1 cm; weight: 70.6 ± 5.5 kg; thirteen right-handed and one left-handed) were all overarm, and all their 30 pitches were analyzed.

In addition, we calibrated three points in the horizontal direction (0.1 m intervals), three in the vertical direction (0.2 m intervals), and three in the home plate-pitcher’s plate direction (0.1 m intervals), giving a total of 27 calibration points for the transformation of the position coordinates. The calibration points were digitized using numerical analysis software (MATLAB, Mathworks, Japan). The position coordinates of the ball and fingers were calculated through direct linear transformation (DLT) using MATLAB. (Please see the S1 File for the results of the validation of the current method.)

We used a fourth-order low-pass digital Butterworth filter with a 60Hz cut-off frequency, which was selected based on the results of residual analysis and qualitative observations of the velocity curves.

### Calculation of parameters

The three-dimensional global orthogonal coordinates were defined as follows: the center of the pitcher’s plate on the mound was considered to be the origin, the x-axis was oriented in the third base direction, the y-axis was in the direction of the home plate, and the z-axis was oriented vertically upward (Fig 1A). The release parameters were defined based on the global coordinate system. Speed was defined as the magnitude of the velocity vector at ball release. The release angle was given by the elevation angle θ_1_ (−90–90◦) and azimuth angle θ_2_ (−90–90◦) of the velocity in polar coordinates (Fig 1B). θ_1_ was the angle between the velocity vector at ball release on the y-z plane and y-axis, and θ_2_ was the angle between the velocity vector on the x-y plane and y-axis. The positive directions of θ_1_ and of θ_2_ were defined as the upward direction and the direction of the third base, respectively.

The variability of the parameters was determined based on the standard deviation (SD) between trials. The index indicating the positional relationship between the ball and fingers, the positional relationship index (PRI) was defined as the time series of the 3D positions of the nails on the index and middle fingers relative to the center of the ball in the global coordinate system (Fig 1C). This index was used for investigating the relationship with the variability of the release angle. The relationship between the variability of the release angle (SDθ_1_ and SDθ_2_) and the PRI for each pitcher was examined using Pearson’s correlational test. The study considered statistical significance at a threshold of P < 0.05. In addition, the ball acceleration was determined by differentiating the velocity, providing additional support for interpretation. The release angle is expected to be influenced by the force exerted on the ball during release, which can be calculated by backtracking from the ball acceleration as the net force acting on the ball.

## Results

### Positional relationship between ball and fingers and release angle

**Fig 2:**
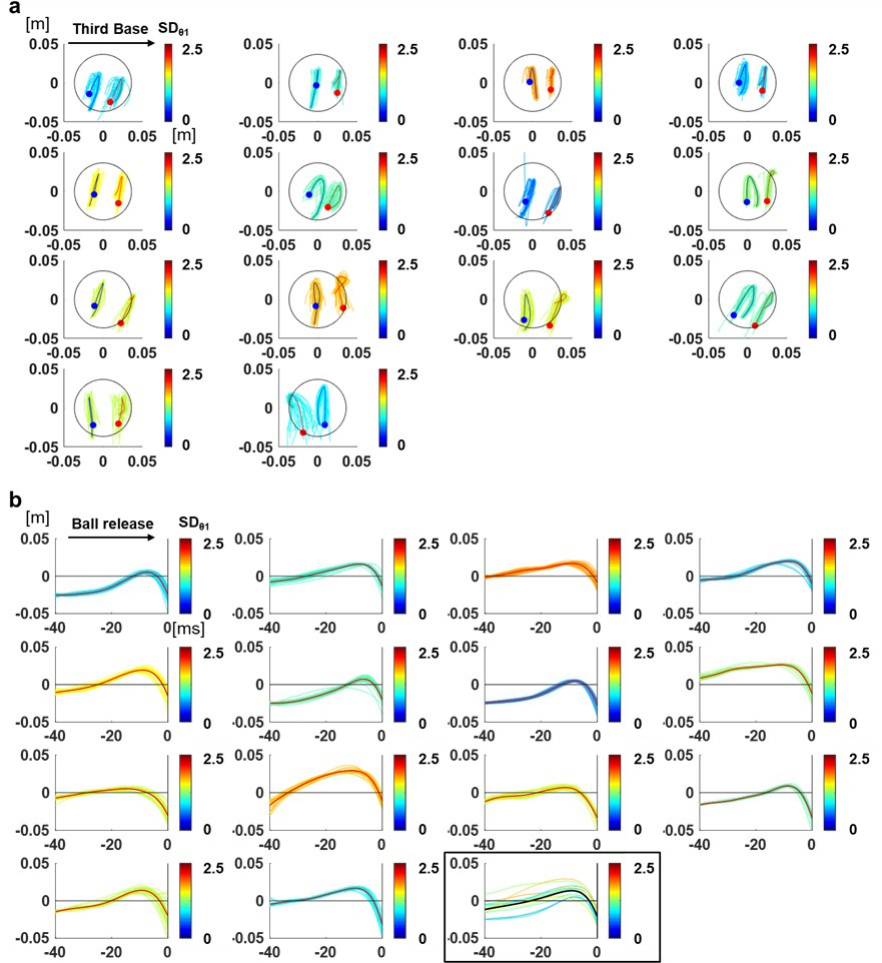
Positional relationship between the ball and fingers. (a) The positions of the nails on the index and middle fingers relative to the center of the ball in the coronal (x-z) plane for each pitcher (-40 to 0ms). The blue and red lines show the averages of 30 trials for the index and middle fingers, respectively, and the colored lines show the value of each trial, whose color corresponds to SDθ_1_. The blue and red plots show the values of the index and middle fingers at ball release. (b) Time series of the z positions of PRI of the middle finger for each pitcher. The red lines show the averages of 30 trials and the colored lines show the value of each trial, whose color corresponds to SDθ_1_. Time = 0 indicates ball release. For the last graph, the thin colored lines show the average of each pitcher, and the bold black line shows the overall average.

Table 1 lists the obtained values of the release parameters including angles, speeds, and positions for all the pitchers. For the left-handed pitchers (L), the values are presented with the left and right sides inverted, and these values were used to calculate the average. Fig 2A shows the time series of the nail positions of the index and middle fingers relative to the center of the ball in the coronal (x-z) plane for each pitcher. The results showed that the movement trajectories of the two fingers were not always parallel and that the movements of the two fingers were not the same.

**Table 1:** Release parameters for each pitcher.

Pitcher	*Speed* [m/s ]	*Angle θ₁* [deg ]	*Angle θ₂* [deg ]	*Position x* [m ]	*Position y* [m ]	*Position z* [m ]
A	32.49 ± 0.85	1.51 ± 0.89	-5.95 ± 0.99	0.47 ± 0.03	1.42 ± 0.05	1.53 ± 0.03
B	29.66 ± 0.70	-0.90 ± 1.08	-7.52 ± 0.84	0.40 ± 0.02	1.77 ± 0.03	1.67 ± 0.01
C	26.82 ± 0.58	1.67 ± 1.81	-3.42 ± 0.55	0.44 ± 0.01	1.29 ± 0.04	1.76 ± 0.02
D	32.19 ± 0.62	-1.53 ± 0.84	-4.08 ± 0.60	0.36 ± 0.02	1.24 ± 0.05	1.75 ± 0.03
E	26.83 ± 0.74	1.36 ± 1.59	-4.16 ± 0.97	0.49 ± 0.04	1.42 ± 0.04	1.72 ± 0.02
F	35.14 ± 0.23	0.72 ± 1.17	-3.11 ± 0.58	0.40 ± 0.02	1.58 ± 0.02	1.54 ± 0.02
G	33.12 ± 0.41	2.81 ± 0.74	-5.72 ± 0.45	1.02 ± 0.02	1.46 ± 0.02	1.46 ± 0.01
H	26.90 ± 0.58	-2.98 ± 1.30	-6.08 ± 1.37	0.39 ± 0.02	1.72 ± 0.03	1.78 ± 0.01
I	29.77 ± 1.53	0.13 ± 1.48	-9.06 ± 0.88	0.38 ± 0.03	1.35 ± 0.11	1.68 ± 0.03
J	32.85 ± 1.05	-7.65 ± 1.75	-2.79 ± 1.97	0.13 ± 0.07	1.44 ± 0.03	1.85 ± 0.02
K	32.53 ± 0.51	2.52 ± 1.46	-2.56 ± 0.74	0.25 ± 0.03	1.49 ± 0.04	1.55 ± 0.02
L	30.11 ± 0.30	0.06 ± 1.67	-4.70 ± 0.76	0.49 ± 0.03	1.57 ± 0.02	1.65 ± 0.02
M	30.81 ± 0.28	0.71 ± 1.44	-3.51 ± 0.98	0.41 ± 0.03	1.75 ± 0.03	1.44 ± 0.01
N(L)	32.52 ± 0.45	-0.44 ± 0.97	-6.14 ± 0.70	0.17 ± 0.03	1.76 ± 0.03	1.76 ± 0.01
Mean	30.84 ± 2.61	-0.14 ± 2.67	-4.91 ± 1.91	0.39 ± 0.25	1.52 ± 0.18	1.65 ± 0.13

Mean release parameters of each pitcher. In the case of left-handed pitchers (with “(L)” next to the pitcher label in the first column), the values are shown with the left and right sides inverted for comparison.

**Fig 3:**
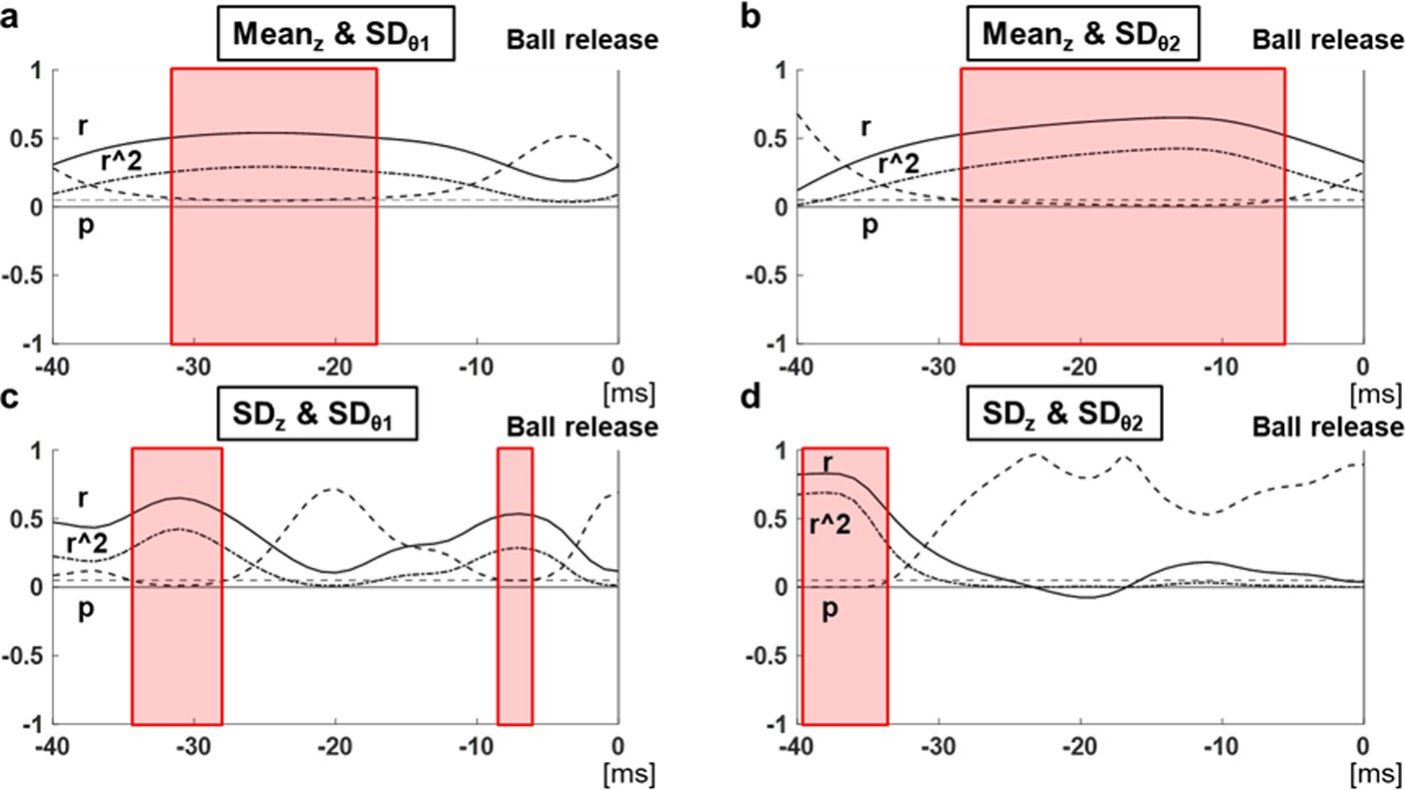
Correlation between the position of the middle finger and SD of the release angles. The graphs show the correlation coefficients r (straight lines) between the (a) average of the z-positions of the middle finger at each time point and SDθ_1_, (b) SD of the z-positions of the middle finger at each time point and SDθ_1_, (c) average of the z-positions of the middle finger at each time point and SDθ_2_, and (d) SD of the z-positions of the middle finger at each time point and SDθ_2_, and their corresponding r^2 (dashed lines) and p-values (broken lines). Time = 0 indicates ball release. The periods bordered in red indicate those in which significant correlation was observed (p < 0.05).

Significant correlation (p < 0.05) was found between the average of z positions of the PRI of the middle finger and SDθ_1_. Fig 2B shows the time series of the z positions of the PRI of the middle finger for each pitcher. Fig 3A shows the correlation coefficients between the average value of the PRI of the middle finger at each time and SDθ_1_, and their p-values. The lower the middle finger position approximately 20-30ms before release was, the smaller the value of SDθ_1_ was. This trend was almost the same for SDθ_2_ (Fig 3B). There were also correlations between the SD of the z positions of the PRI of the middle finger at the earlier and later time periods (approximately 30-35ms and 7-9ms before release) and SDθ_1_, and between the SD of the z positions of the PRI of the middle finger at the earlier short time periods (approximately 35-40ms before release) and SDθ_2_ (Fig 3C and 3D). The lower the variability of the middle finger positions at these time periods was, the smaller the variability in the release angles was. Table 2 shows the summary of the correlation coefficients between the PRI of the middle finger at each time and SDθ_1_ or SDθ_2_. Meanwhile, no significant correlation was found between the average or SD of the x values of the PRI of the middle finger or the x values and z positions of the PRI of the index finger and SDθ_1_ or SDθ_2_. Here, 30-35ms and 7-9ms before release, during which correlation was found with the SD of the positional relationship, were defined as Phase 1 and Phase 3, respectively, and 20-30ms before release, during which correlation was found with the average of the positional relationship, was defined as Phase 2 for convenience.

**Table 2:** Correlation coefficients between the variability of the release angle and the positional relationship index.

Time	*Mean*_*z*_ *& SD*_*θ1*_	*Mean*_*z*_ *& SD*_*θ2*_	*SD*_*z*_ *& SD*_*θ1*_	*SD*_*z*_ *& SD*_*θ2*_
-40	0.308	0.121	0.475	0.823
-39	0.345	0.177	0.459	0.828
-38	0.379	0.230	0.441	0.831
-37	0.409	0.279	0.435	0.826
-36	0.435	0.324	0.457	0.795
-35	0.456	0.363	0.503	0.717
-34	0.474	0.399	0.557	0.600
-33	0.488	0.430	0.608	0.482
-32	0.501	0.458	0.643	0.381
-31	0.511	0.481	0.652	0.299
-30	0.520	0.502	0.636	0.232
-29	0.527	0.520	0.599	0.178
-28	0.533	0.535	0.547	0.135
-27	0.537	0.549	0.485	0.102
-26	0.540	0.561	0.415	0.073
-25	0.541	0.572	0.340	0.047
-24	0.541	0.582	0.267	0.019
-23	0.538	0.592	0.200	-0.009
-22	0.535	0.601	0.146	-0.037
-21	0.530	0.610	0.113	-0.061
-20	0.524	0.618	0.106	-0.075
-19	0.517	0.625	0.126	-0.074
-18	0.510	0.632	0.168	-0.053
-17	0.503	0.638	0.220	-0.012
-16	0.495	0.643	0.267	0.041
-15	0.486	0.648	0.296	0.095
-14	0.474	0.651	0.309	0.136
-13	0.459	0.653	0.317	0.162
-12	0.439	0.651	0.341	0.178
-11	0.413	0.644	0.391	0.183
-10	0.382	0.633	0.453	0.171
-9	0.346	0.615	0.501	0.147
-8	0.306	0.593	0.529	0.126
-7	0.267	0.566	0.536	0.111
-6	0.232	0.536	0.524	0.102
-5	0.205	0.504	0.488	0.098
-4	0.190	0.472	0.415	0.092
-3	0.190	0.437	0.303	0.078
-2	0.207	0.401	0.195	0.057
-1	0.244	0.364	0.132	0.041
0 (release)	0.298	0.327	0.116	0.039

Correlation coefficient r between the variability of the release angle and the positional relationship index at each time point. In the shaded time periods, significant correlation (p < 0.05) was found.

### Positional relationship between ball and fingers and acceleration

**Fig 4:**
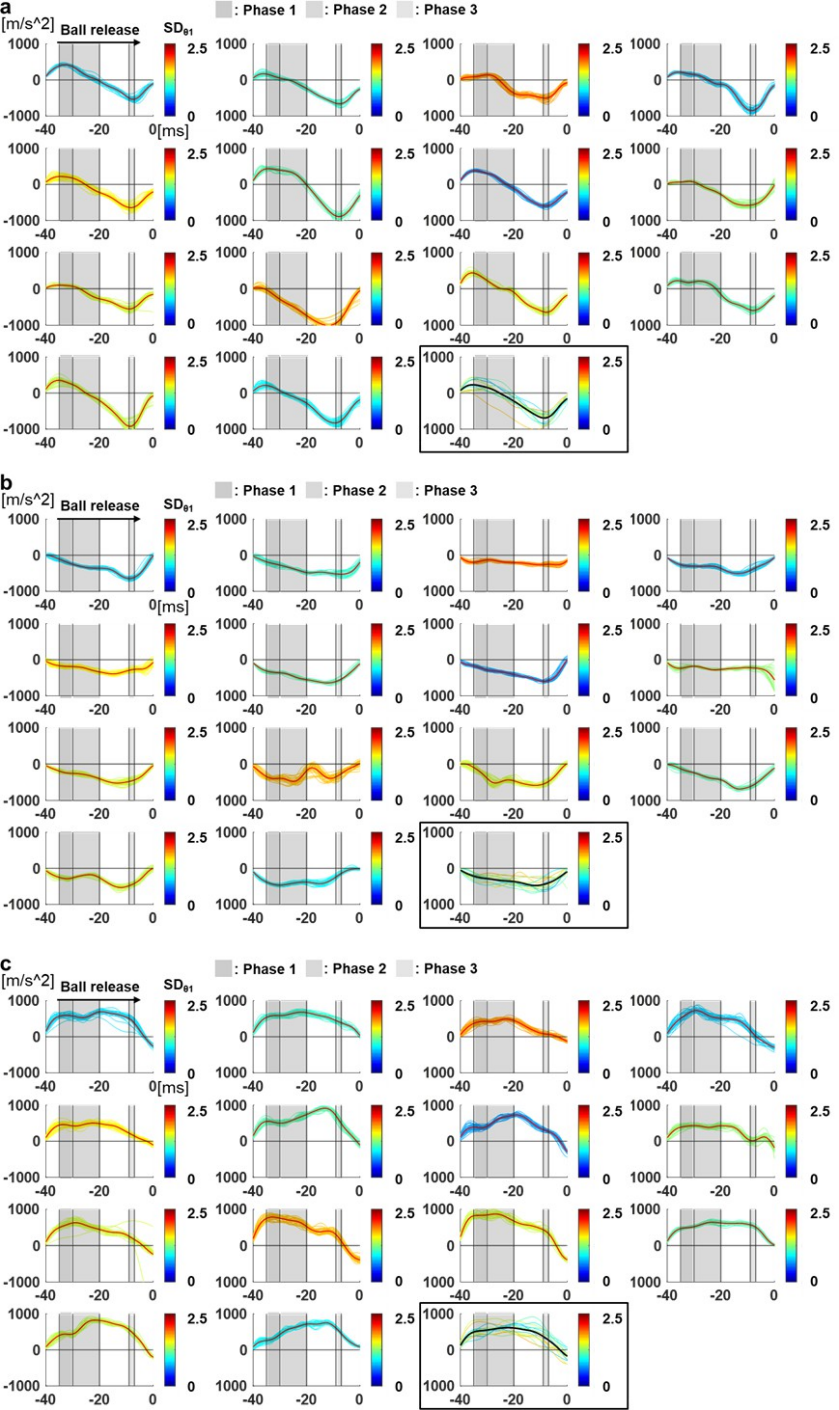
Time series of the acceleration of the ball. (a) Time series of the z-acceleration of the ball for each pitcher. The red lines show the averages of 30 trials and the colored lines show the value of each trial, whose color corresponds to SDθ_1_. Time = 0 indicates ball release. For the last graph, the thin colored lines show the average of each pitcher, and the bold black line shows the overall average. Points in which the acceleration changes from upward to downward were observed 20-30ms before release (average: 22.81ms before release), where correlation was found between the average z-position of the middle finger and SDθ_1_. In addition, peaks of the upward acceleration (average: 33.63ms before release) and downward acceleration (average: 8.88ms before the release) were observed in 30-35ms and 7-9ms before release, respectively, where correlations were found between the SD of the z-positions of the middle finger and SDθ_1_. (b) Time series of the x-acceleration of the ball for each pitcher. The red lines show the averages of 30 trials and the colored lines show the value of each trial, whose color corresponds to SDθ_1_. Time = 0 indicates ball release. For the last graph, the thin colored lines show the average of each pitcher, and the bold black line shows the overall average. The time series change of the x-acceleration of the ball was less than that of the z-acceleration and there were no clear peaks for most pitchers. (c) Time series of the y-acceleration of the ball for each pitcher. The red lines show the averages of 30 trials and the colored lines show the value of each trial, whose color corresponds to SDθ_1_. Time = 0 indicates ball release. For the last graph, the thin colored lines show the average of each pitcher, and the bold black line shows the overall average. The time series change of the y-acceleration of the ball was less than that of the z-acceleration and there were no clear peaks for most pitchers.

To determine the reason for the correlation between the PRI and SDθ_1_ in the above-mentioned time periods, we calculated the time series change in the acceleration of the ball. Fig 4A shows the time series of the z-acceleration of the ball for each pitcher. We compared the time series change of the z-acceleration of the ball and the time periods in which correlations between the PRI and SDθ_1_ were found. In Phase 2, a correlation was found between the average of the z-positions of the middle finger and SDθ_1_; this region contained points where the acceleration changed from upward to downward (average: 22.81 ± 5.42 ms before release). In addition, Phase 1 and Phase 3, in which correlations were found between the SD of the z-positions of the middle finger and SDθ_1_, contained peaks of the upward acceleration (average: 33.63 ± 1.21 ms before release) and downward acceleration (average: 8.88 ± 2.05 ms before release), respectively. These results indicate that with lower middle finger position when the acceleration direction changes and decreasing variability in the middled finger position at the peaks of the acceleration, the value of SDθ_1_ decreases. However, the time series change of the x acceleration of the ball was smaller than that of the z acceleration, and there were no clear peaks for most pitchers (Fig 4B).

## Discussion and implication

### Positional relationship between ball and fingers and release angle

The mean vertical and horizontal release angles in this study were -0.14 ± 2.67◦ and -4.91 ± 1.91◦, respectively, whereas Kusafuka et al. (2021) and Nasu et al. (2021) reported mean vertical and horizontal release angles of 1.37 ± 2.43◦ and -3.72 ± 0.87◦, and -1.18 ± 1.09◦ and -3.41 ± 0.85◦, respectively. The values of the release angles in this study differ only slightly from those of previous studies, indicating that the proposed method obtained the data successfully. In addition, the values of the other parameters were similar to those reported previously.

We examined the time series change of the positional relationship between the ball and fingers to clarify the relationship with the variability of the release angle, which constitutes the primary factor influencing the variability of pitch locations in fastball pitching [7]. As a result, low middle finger positions when the vertical acceleration direction changed and low variability in the middle finger position at the vertical acceleration peaks decreased the variability of the elevation release angle. Although, given the laws of physics, the release angle could be determined based on the force with which the ball was released, no correlation was found between the positional relationship of the ball and fingers at ball release and the variability of the release angle. The presumed reason was that the acceleration (and also the net force acting on the ball) was almost zero during ball release (Fig 4A). This result is in agreement with that of a previous study [13]. If the force is small, it will not be reflected in the velocity angle unless it is maintained for a significant period. Therefore, the effect of the variability of state at the acceleration peaks (Phase 1 and Phase 3), where a large force occurred, on the variability of the release angle would be large. However, as the time series change of the horizontal acceleration was small compared to that of the vertical acceleration (Fig 4B), the variability of state at a particular point was not considered to affect the variability of the azimuth velocity angle.

The force is determined by three elements: magnitude, direction, and point of action. The results suggest that the variability of the positional relationship between the ball and fingers, i.e., the action point, influences the variability of the release angle. With respect to the effect of the magnitude, no correlation was found between the variability of magnitudes of the peak accelerations in Phase 3 and SDθ_1_. This suggests that the variability of the action point, rather than the variability of the magnitude of the force itself, influences the variability of the release angle.

Moreover, the lower middle finger positions in Phase 2 could have reduced the variability of the middle finger positions in Phase 3. When manipulating an object, the controlled object moving in the direction of the grasping force (on the line connecting the index/middle finger and the thumb) is considered to have a higher positional stability against the hand and fingers, with robust resistance against spatial variability [16, 17]. Because the movement direction of the ball was upward (20–40°) in Phase 2, the grasp stability was considered high when the middle finger position was low and grasping force was directed upward. Therefore, the lower middle finger positions in Phase 2 (before the acceleration peaks) could have reduced the variability of the middle finger positions in Phase 3 (the acceleration peaks just before release) and consequently, reduced the variability of the release angle. This may be accomplished by dorsiflexing the hand as much as possible. Previous research has shown that some of the best-performing throwers have robust hand trajectories against the variability during release time that compensate for the timing error [18]. The results of the current study suggest that pitchers with low variability in the pitch location perform robust body movement against the spatial variability of ball movement.

### Implications for biomechanics and motor control

In control engineering, it is necessary to consider even the minute aspects of the fingertips in handling objects [19]. However, in biomechanics, fingers are often treated as an integral part of the palm, and accurate studies on finger movements in human whole-body movements have been limited in comparison to those on other body-part movements. This is because it is difficult to accurately measure finger movements owing to their size, narrow camera angle view, and ease of removing the markers in the conventional method involving the acquisitions of a marker attached to the body, with optical cameras at high-speed, in particular. This study attempted to obtain detailed finger movement characteristics using automatic image recognition technology based on deep learning developed in recent years and examined the importance of fingers as the contact point between the body and an object in human motor control. As this method does not require markers attached to the body, data acquisition is possible without interfering with movement or sensation. As a result, it was revealed that the movements of index and middle fingers were not identical. An advanced method for analyzing finger movements, which has not been focused on in the past, would be significant in the field of biomechanics because it could reveal subtle movements that are difficult to observe with the naked eye.

The results suggest that people skilled in motor control employ movements that are robust against the spatial variability, i.e., the contact/release of objects is also a motor control skill. Contact between the body and object could be involved in motor control, not only in terms of the mechanics but also in terms of sensing. Subtle sensory control of the fingertips is often important in skilled human movements, and it has been shown that the sensory information obtained from the fingers influences the accuracy of the resulting performance [20–22]. As there is a limit to the speed of neural conduction, it may be reasonable to assume that the state in the phases preceding ball release (not at ball release) is important, although whether movement control in less than 30 ms is possible cannot be clarified through this study. The findings from a biomechanics perspective, which indicate the importance of considering in more detail the role of fingers in movements for controlling an object with the hands and fingers, are significant for motor control.

### Limitations and future issues

With the recent advances in science and technology, remarkable progress has been made in measurement equipment and data analysis technology. It is remains challenging to measure the fine forces in fast human whole-body movements accurately and without interfering with the movement or sensation, although only a study by (Kinoshita et al., 2017) [23] has succeeded. In the study, an instrumented ball in which a tri-axial force transducer with a cable was installed was developed for direct measurement of ball reaction force by individual fingers during pitching. In the present study, the force was analyzed by estimating the acceleration through inverse calculation from the ball movements. As correlation was found only in the middle finger movement, the force acting on the ball from the middle finger might be larger than that of the index finger. If a measurement technology (e.g., a sensor attached to the surface of the ball) that enables the easy acquisition of force is more general and spread in future, it can be combined with the positional relationship found in this study to obtain details on the process determining ball movement. In addition, although the position of the fingertips relative to the center of the ball was considered as the contact point in this study, there could be multiple points of contact between the finger and the ball, not only the fingertips. To consider all the actual points of contact between the finger and ball, it is still important to obtain the exact forces applied to the ball.

This study has certain limitations. The participants always threw at the same target position, and there was no batter, which was different from the environment in actual baseball games. The level of the participants was limited to university baseball. It should also be noted that the result may be specific to the overarm throw style. In future, investigation with more participants having various skill levels and pitching styles in more practical settings is required.

## Conclusion

This study focused on the time series change of the positional relationship between the ball and fingers and examined its relationship with the variability of the release angle, which is the main factor affecting the variability of pitch location in baseball pitching, to clarify the characteristics of body movements for accuracy in one of the specific human motor skills. To acquire detailed finger movements without interfering with the movement or sensation, we used an automatic image recognition technology based on deep learning. As a result, as the middle finger positions were low when the vertical acceleration direction changed and its variability at the peaks of the vertical acceleration decreased, the variability of the elevation release angle decreased. The lower middle finger positions before the acceleration peaks could have reduced the variability of the middle finger positions at the acceleration peaks where a large force occurs immediately before release, and consequently reduced the variability of the release angle. This suggests that pitchers with low variability of the release angle positioned their fingers robustly against the spatial variability of ball movement, i.e., the contact/release of an object is also a motor control skill.

## Supporting information

S1 File (DOCX)
