# Supplementary material for: Positional relationship between ball and fingers for accurate baseball pitching
Source: PLoS One. 2023 Dec 19;18(12):e0290042. doi: 10.1371/journal.pone.0290042 (PMC10729979; doi:10.1371/journal.pone.0290042)
Supplement: S1 File — (DOCX) [file pone.0290042.s001.docx]

**Supplementary Materials**

We compared the position of the markers of ball and fingers calculated using the method proposed this study with those obtained through the conventional optical motion capture using skin markers. Reflective markers were attached to the same position of the throwing hand, whose coordinates were obtained using our method and six reflective markers were attached to the ball for calculating the coordinates of its center. Two same high-speed cameras as in the study (DSC-RX10M4, SONY, Japan) and an optical motion capture system with ten cameras (MAC3D System, Motion Analysis Corporation, USA) recorded the three-dimensional coordinates of the positions of the reflective markers at the same sampling rate (960 Hz). The data of high-speed cameras and an optical motion capture system were synchronized. Figure S1a, b show the positions of the index and middle fingers and the ball in a typical pitch (from 40 frames before ball release to ball release) calculated through the proposed method and optical motion capture, respectively; Figure S1c shows the plots determined through the proposed method and the optical motion capture plots of the x, y, z positions of the nail on the index and middle fingers relative to the center of the ball (the index indicating the positional relationship between the ball and fingers) in a pitch with an ideal line (45◦ straight line). R squared was greater than 0.9 throughout. The root mean square error in a pitch (from 40 frames before ball release to ball release) was 0.008 ± 0.003 m in index finger relative to the center of the ball and 0.009 ± 0.003 m in middle finger relative to the center of the ball. This trend didn't change even for several pitches. Thus, the performance of the proposed method is excellent and it can calculate the position of ball and fingers correctly.

Figure S1.


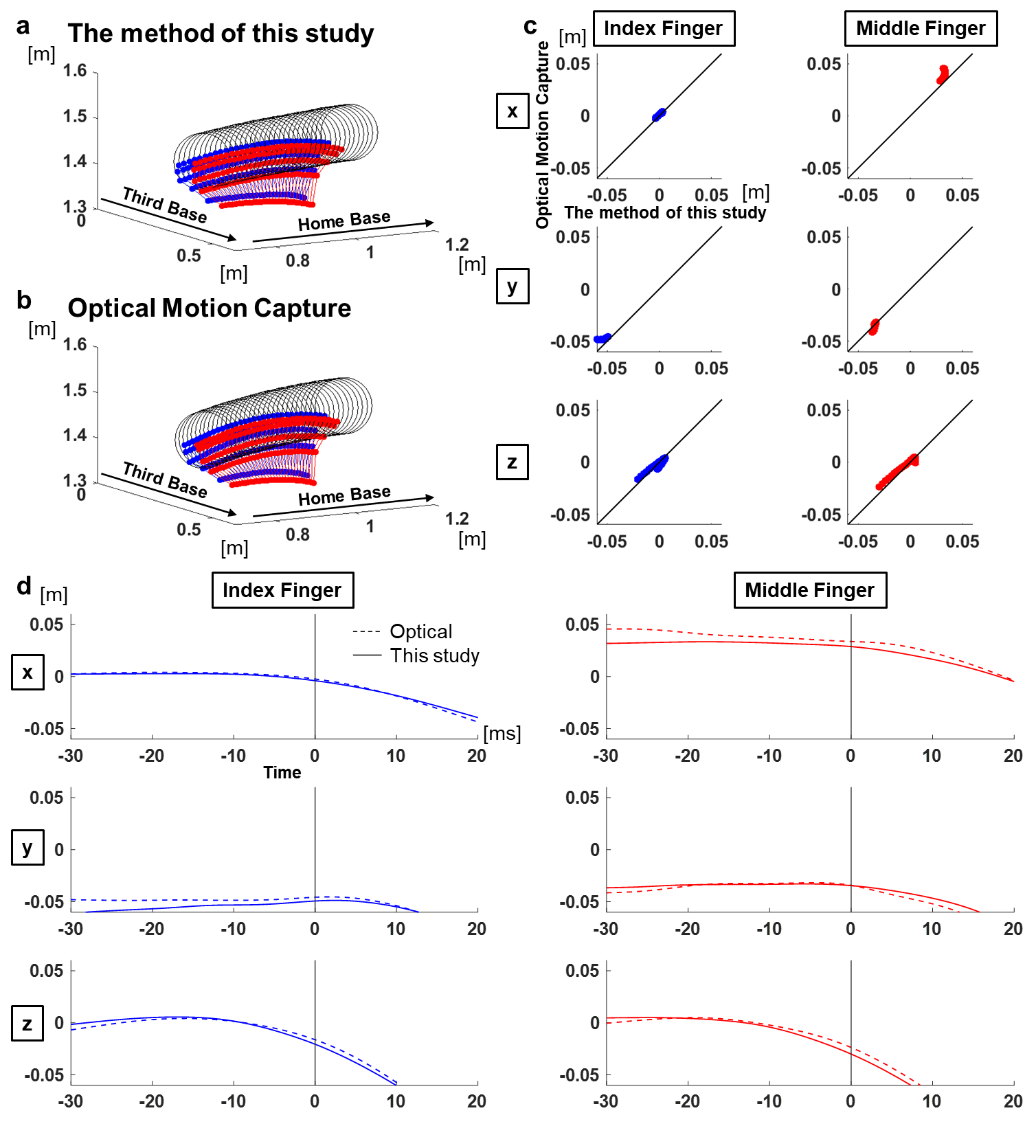


Figure S1. Comparison the position of the markers calculated through the proposed method and those determined through optical motion capture.

(a) Graph of the positions of the index and middle fingers and the ball in a typical pitch (from 40 frames before ball release to ball release) calculated through the proposed method. The blue and red plots and lines show the positions of the index and middle finger, respectively and the black circles show the ball positions.

(b) Graph of the positions of the index and middle fingers and the ball in a typical pitch (from 40 frames before ball release to ball release) calculated trough optical motion capture. The blue and red plots and lines show the positions of the index and middle finger, respectively and black circles show the ball positions.

(c) Graphs of the proposed method vs the optical motion capture plots of the x, y, z positions of the nail on the index and middle fingers relative to the center of the ball (index indicating the positional relationship between the ball and fingers) in a typical pitch (from 40 frames before ball release to ball release) with an ideal line (45 ◦ straight line). The blue and red plots show the positions of the index and middle finger, respectively.

(d) Graphs of the proposed method (solid line) and the optical motion capture (break line) time-series profiles of the x, y, z positions of the nail on the index and middle fingers relative to the center of the ball (index indicating the positional relationship between the ball and fingers) in a typical pitch (from 40 frames before ball release to ball release). The blue and red lines show the positions of the index and middle finger, respectively.
